# Analysis of genetic instability induced by radon exposure in iron mine processing workers in Shandong Province, Northern China

**DOI:** 10.3389/fpubh.2024.1452730

**Published:** 2024-12-13

**Authors:** Lianying Fang, Fangfang Wu, Hao Sun, Weiguo Li, Dianjun Hou, Ya Ma

**Affiliations:** School of Preventive Medicine, Shandong First Medical University (Institute of Radiation Medicine, Shandong Academy of Medical Sciences), Jinan, Shandong, China

**Keywords:** randon, genetic instability, miners, occupational health, ionizing radiation

## Abstract

**Background:**

Radon, a colorless and odorless radioactive gas, poses serious health risks. It is the second leading cause of lung cancer and notably increases lung cancer risk in smokers. Although previous epidemiological studies have mainly examined lung cancer rates in miners, the effects of radon on genomic stability and its molecular mechanisms are not well understood.

**Methods:**

This study evaluated chromosomal aberrations (CA) and cytokinesis-block micronucleus (CBMN) in miners’ lymphocytes, investigating the relationship between cytogenetic damage and variables such as exposure duration and age. Additionally, gene expression profiles were compared between radon-exposed miners and a control group to identify genes involved in DNA damage repair.

**Results:**

We observed a significant increase in CA and CBMN among underground miners. Gene expression analysis showed 14 genes were upregulated and four downregulated in the exposed group compared to controls.

**Conclusion:**

These findings indicate a strong link between high radon exposure and genomic instability in miners. Improved monitoring of work environments and stronger protective measures are critical to safeguarding miners’ health.

## 1 Introduction

Lung cancer remains the leading cause of cancer-related deaths worldwide, with both incidence and mortality rates increasing annually (1). In China, it poses a severe public health challenge, as it is the leading cause of cancer mortality for both genders, contributing significantly to the societal burden (1). After tobacco smoke, radon is the second most important cause of lung cancer (2, 23). Studies show that radon and smoking have a multiplicative effect on lung cancer risk, with radon acting as an independent risk factor that heightens the danger for smokers (3). And the current findings provide evidence for an increased lung cancer risk at low radon exposures or exposure rates, but the observed risk is lower. And the previous research has also shown that the possibility of a hormetic effect on lung cancer at low radiation doses cannot be excluded. An example is a case–control study of the lung cancer risk from residential radon exposure, in which the results indicated that for radon levels up to and somewhat exceeding the EPA’s action level of 4 picocuries/L of air (approximately 150 Bq/m^3^), the lung cancer odds ratio was <1, implicating negative values for excess relative risk, which translated into a reduction in lung cancer risk (4, 5).

Radon is a naturally occurring, colorless, and odorless radioactive gas produced by the decay of uranium and thorium in soil and rock (6). It is the largest single contributor to natural radiation exposure, with a half-life of 3.83 days (7). Of the three naturally occurring isotopes—^222^Rn, ^220^Rn, and ^219^Rn—only ^222^Rn and ^220^Rn significantly affect human exposure (8). Radon tends to accumulate in enclosed spaces like mines, basements, and caves, making it a key factor in annual radiation exposure (9). When inhaled, radon particles can attach to lung tissues, causing ongoing radiation damage that can spread to the bloodstream, leading to broader health risks (10). The international agency for research on cancer (IARC) classifies radon as a class I carcinogen, confirming it as the second leading cause of lung cancer after smoking (11).

Shandong Province in Northern China, rich in mineral resources, has a large mining workforce. However, limited awareness of radiation risks and insufficient safety policies put underground miners at significant risk of radon exposure. While past research, including studies on uranium miners in Germany and tin miners in China, has primarily linked radon exposure to lung cancer, the specific effects on genomic stability and molecular mechanisms remain unclear (12, 13). This study investigated 313 miners from an iron mine in Shandong Province. Radon exposure was monitored quarterly using CR-39 nuclear track detectors. Peripheral blood samples were collected to examine changes in unstable chromosome aberrations (CAs), cytokinesis-block micronuclei in lymphocytes, and gene expression through RNA sequencing (RNA-seq). This study aimed to extend previous findings by providing a detailed analysis of the health risks posed by radon exposure in underground miners.

## 2 Methods

### 2.1 Study population

This cohort study was conducted among miners at a non-uranium mine in Shandong Province, China. The exposed group included 189 males who worked underground and had no prior occupational exposure to other hazards. Daily activities involved underground rock drilling and blasting, with potential exposure to radon. The reference group included 124 men employed above ground, primarily in management positions. Monitoring of radon exposure in the underground miners was performed every 3 months. The dose detected in was the personal committed effective dose, the track density of alpha particles was performed using solid state nuclear track detectors (CR-39 detector), and then the cumulative exposure of individual radon was calculated. Finally, the personal committed effective dose was calculated based on the conversion factor from individual exposure to committed effective dose. Personal dosimetry results for the exposed group ranged from 0.25 to 7.29 mSv/a, with an average of 4.72 ± 2.68 mSv/a. And the radon concentration in the underground miners’ workplace was 270–3,945 Bq/m^3^, with a median value of 2,108 Bq/m^3^. Informed consent was obtained from all participants, and the inclusion criteria were established as follows: males employed for at least 1 year and without acute infectious diseases, malignant tumors, or significant family histories of genetic disorders.

### 2.2 Chromosome aberration analysis

Chromosome aberration was quantified following established methods (14). Peripheral blood was incubated in RPMI 1640 medium containing 10% fetal bovine serum, 150 μg/mL phytohaemagglutinin (PHA), and 0.06 μg/mL colchicine under 5% CO_2_ at 37°C for 48 h. Lymphocytes were then harvested, subject to hypotonic treatment, fixed, and prepared for Giemsa staining. A minimum of 200 metaphase cells with well-spread chromosomes were analyzed for each individual in both groups. Images of the metaphase chromosomes were captured using the Metafer Automated Slide Scanning Platform (Zeiss, Germany). The types of chromosomal aberrations recorded included dicentric chromosomes (dis), ring chromosomes (r), and acentric fragments (ace), which were summed to determine the total number of CAs.

### 2.3 Cytokinesis-block micronucleus assay

Cytokinesis-block micronucleus aberrations were quantified following established protocols (15). Briefly, peripheral blood samples were cultured in RPMI 1640 medium at 37°C for 44 h, after which 6 μg/mL Cytochalasin-B was added, and the samples were incubated for an additional 24 h. Subsequently, cells were harvested, fixed, and stained for 10 min with 5% Giemsa solution. Images of binucleated lymphocytes were captured using a fully automated micronucleus scanning and analysis system (Beion, China). An AI system automatically identified CBMN, which were then subjected to manual review. For each sample, 1,000 binucleated cells were analyzed to determine CBMN frequency.

### 2.4 Gene expression analysis

Gene expression profiling data were obtained from 12 donors, categorized into four groups based on the duration of underground service: 0 years, <10 years, 10–20 years, and > 20 years. Total RNA was extracted from retinal explants using TRIzol reagents (Invitrogen, Carlsbad, CA) and treated with RNase-free DNase I (New England Biolabs, Beverly, MA) to remove genomic DNA. Eighty-two key genes from the Human DNA Damage Signaling Pathway were simultaneously analyzed using the RT^2^ Profiler PCR array plate, adhering to the manufacturer’s protocol. Sequencing libraries were prepared with the NEBNext Ultra Directional RNA Library Prep Kit for Illumina (New England Biolabs). All analyses were performed on BMKCloud.^1^ A heatmap was generated using the R package gplots and polished with Adobe Illustrator. Pearson’s correlation coefficient was employed to evaluate the dose-dependent expression of mRNA for the targeted genes, resulting in the identification of two main clusters corresponding to genes that were either upregulated or downregulated.

### 2.5 Statistical analysis

Statistical analyses were conducted using GraphPad Prism 5.0 and SPSS version 26.0 (SPSS, Chicago, Illinois). Demographic characteristics, CA, and CBMN data were analyzed using a cross-tabulation Chi-square test for both the exposure and control groups. The statistical significance of CA and CBMN results between the exposure and reference groups was assessed using a Student’s *t*-test. Interactions between smoking habits and semen quality were analyzed using a two-way ANOVA. A *p* value of less than 0.05 was considered statistically significant.

## 3 Results

### 3.1 Demographic characteristics of the study populations

The study included 313 miners from an iron mine in Shandong Province, China, as detailed in Table 1. The exposed group comprised 189 underground miners (average age 44.1 years), and the control group included 124 above-ground miners (average age 41.7 years). Crosstab analysis revealed no statistically significant differences in demographics—age, smoking index, drinking status, or radon awareness—between the groups (*p* > 0.05).

**Table 1:** Demographic characteristics of study participants by group [*n* (%), the percentage of the total].

Covariate	Exposed group (*n* = 189)	Reference group (*n* = 124)	*p* value (χ^2^)
Randon exposure duration (years)			
<10	24 (12.7)	0	
10–20	78 (41.3)	0	
>20	87 (46.0)	0	
Age (years)			0.947 (0.011)
20–29	10 (5.3)	18 (14.5)	
30–39	38 (20.1)	38 (30.6)	
40–49	90 (47.6)	30 (24.2)	
≥50	51 (27.0)	38 (30.6)	
Average	44.1	41.7	
Smoking index			0.083 (3.272)
Yes	99 (52.4)	52 (41.9)	
No	90 (47.7)	72 (58.1)	
Dinking status			0.679 (0.231)
Yes	44 (23.2)	26 (21.0)	
No	145 (76.8)	98 (79.0)	
Radon awareness			0.299 (1.263)
Yes	47 (24.9)	38 (30.6)	
No	142 (75.1)	86 (69.4)	

### 3.2 Comparison of CA and CBMN rates between groups

To assess the relationship between DNA aberrations and prolonged radon exposure, the exposed group was divided into three subgroups by exposure duration (<10 years, 10–20 years, and > 20 years). DNA aberrations, indicated by CA and CBMN rates, are presented in Tables 2, 3. The exposed group exhibited a total of 860 CAs, significantly higher than the 223 observed in the control group, with CA frequencies of 2.3% in the exposed group versus 0.9% in controls (*p* < 0.05). Similarly, CBMN rates were elevated in underground workers compared to controls (24.7‰ vs. 13.6‰, *p* < 0.05; Table 3). Further analysis (Table 4) showed that miners exposed for more than 20 years had significantly higher CA and CBMN rates than those with ≤10 years of exposure, suggesting a clear association between longer exposure and increased DNA aberration rates.

**Table 2:** CA in lymphocytes of the exposure and control groups.

Group	Subjects	Cells	Total aberrations	CA rate	χ^2^	*p*
Exposure	189	37,800	860	2.3%	16.0	<0.001
Reference	124	24,800	223	0.9%		

**Table 3:** CBMN frequency in lymphocytes of the exposure and control groups.

Group	Subjects	Cells	Total micronucleus	CBMN rate	χ^2^	*p*
Exposure	189	189,000	3,943	24.7‰	5021.3	<0.001
Reference	124	124,000	1,399	13.6‰		

**Table 4:** Relationship between working duration and CBMN and CA rates in the exposure group.

Group	Subjects	Chromosome			CBMN		
		Cells	Total aberrations	CA rate	Cells	Total micronucleus	CBMN rate
≤10	24	4,800	160	1.7%	24,000	338	14.1‰
10–20	78	15,600	310	2.0%^a^	78,000	1,466	18.8‰^a^
>20	87	17,400	44	2.5%^a^^,^^b^	87,000	2,305	26.5‰^a^^,^^b^

CA, Chromosome aberration; CBMN, Cytochalasin binuclear micronuclei.

a Compared with ≤ 10 group, *p* < 0.05.

b Compared with 10–20 group, *p* < 0.05.

### 3.3 Age-related comparisons of CA and CBMN among underground miners

To assess the relationship between age and DNA aberrations in underground miners, CA and CBMN rates were analyzed across four age groups (20–29, 30–39, 40–49, and ≥ 50 years) in the exposed group. As shown in Tables 5, 6, CA and CBMN frequencies significantly increased with age (*p* < 0.05).

**Table 5:** Interaction between CA and age in the exposure group.

Group	Subjects	Cells	Total aberrations	CA rate	χ^2^	*p*
20–29	10	2,000	30	1.5%	20.3	<0.001
30–39	38	7,600	140	1.8%		
40–49	90	18,000	760	4.2%		
≥50	51	10,200	590	5.8%		

**Table 6:** Interaction between CBMN and age in the exposure group.

Group	Subjects	Cells	Total micronucleus	CBMN rate	χ^2^	*p*
20–29	10	10,000	121	12.1‰	205.3	<0.001
30–39	38	38,000	567	18.9‰		
40–49	90	90,000	1,989	22.1‰		
≥50	51	51,000	1,397	27.4‰		

### 3.4 Relationship between smoking habit and radon exposure in both groups

**Figure 1:**
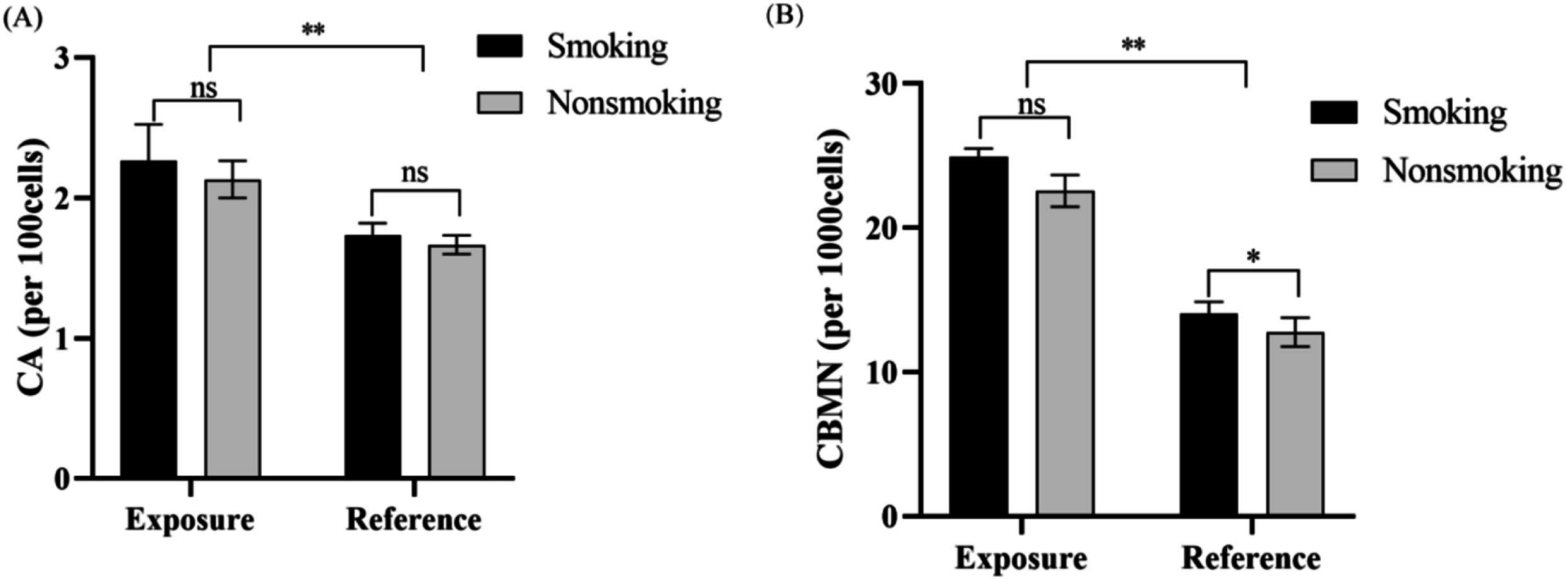
The influence of smoking habits on radon exposure in underground miners. **(A)** CA in lymphocytes from smoking and non-smoking miners. No significant difference in CA rates was observed between the two groups in both the exposure and control groups. **(B)** CBMN rates in lymphocytes from smoking and non-smoking miners. While the CBMN rate was higher in smoking miners in the control group, no significant difference was noted between the two groups in the exposure group. Both CBMN and CA rates were significantly elevated in the exposure group compared to the control group (**p < 0.01; *p < 0.05).

To explore the potential link between smoking and chromosomal aberrations, we categorized both the exposed and reference groups into smokers and non-smokers. In the reference group, smokers exhibited a significantly higher micronucleus rate than non-smokers. In contrast, in the exposed group, no significant differences in chromosomal aberration or micronucleus rates were found between smokers and non-smokers (Figures 1A,B). These results indicate that smoking does not interact with radon exposure among underground miners.

### 3.5 Gene expression profiling in peripheral blood

**Figure 2:**
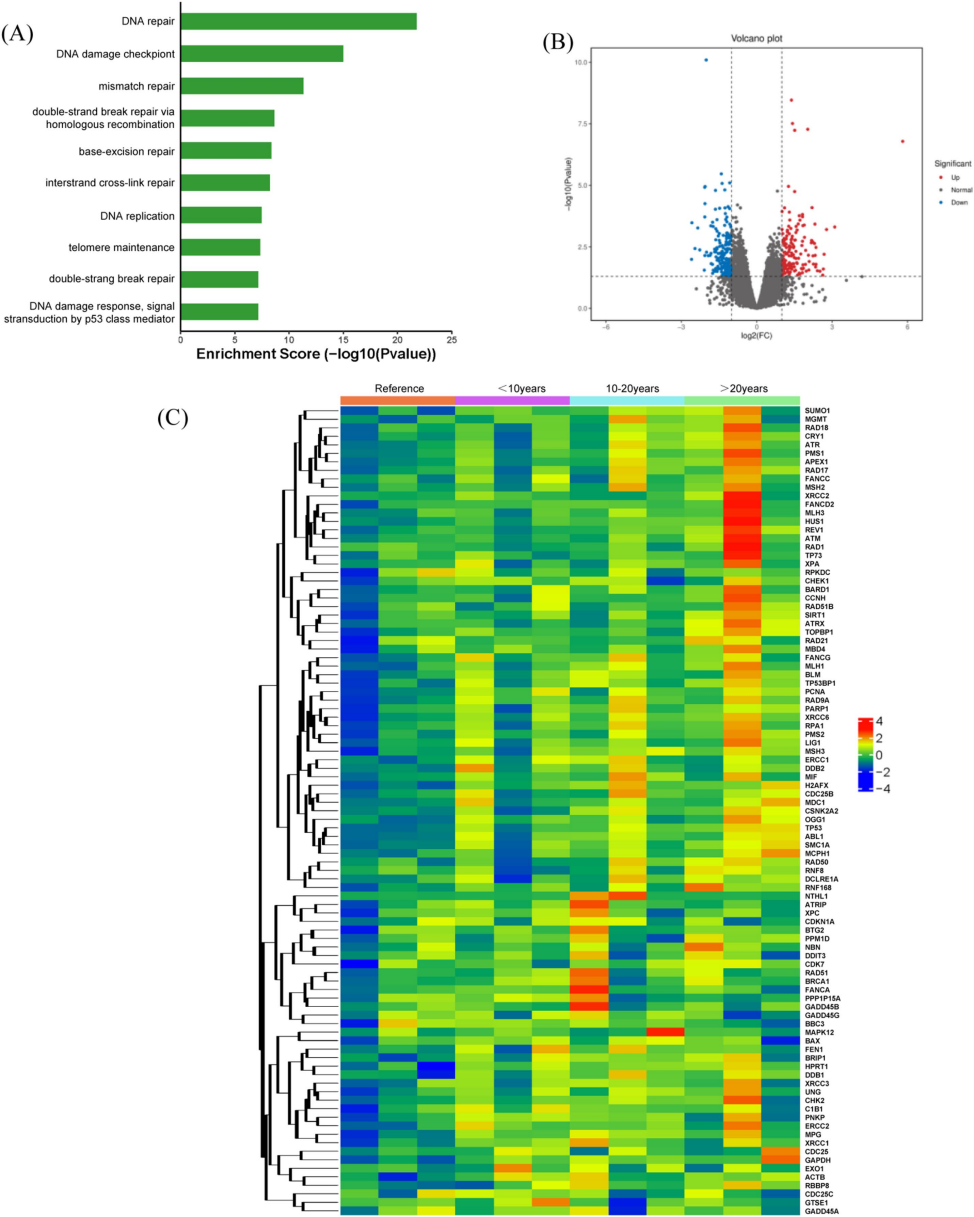
Gene expression analysis in miners from exposure and control groups. **(A)** Gene enrichment analysis illustrating biological processes and pathways associated with gene functions in underground miners. **(B)** Volcano plots displaying differentially expressed genes between the exposure and control groups, with a significance threshold of *p* < 0.05. **(C)** Heat map illustrating gene expression responses to radon exposure in both underground and above-ground miners. Red and blue dots indicate genes with >2-fold up-regulation and down-regulation, respectively.

Previous studies have shown significant DNA damage in radon-exposed miners. To explore the mechanisms behind radon-induced DNA damage in peripheral blood lymphocytes, nine underground miners (stratified by service duration) were selected as the study group, with three above-ground miners as controls. Gene expression related to DNA integrity was analyzed using the RT^2^ Profiler PCR array, followed by bioinformatics and statistical analyses to identify key genes associated with radon-induced damage. Figure 2 presents a heat map of genes with significant expression changes (over 2-fold up- or downregulation). Fourteen genes, including *ATM*, *ATR*, *ATPX*, *RAD1*, *RAD18*, *PMS1*, *XRCC2*, *FANCD2*, *MLH3*, *HUS1*, *TP73*, *XPA*, *BARD1*, and *CCNH*, were upregulated, while *BAX*, *DDIT3*, *BBC3*, and *CDC25C* were downregulated in underground miners (Figure 2C). Gene ontology (GO) analysis further indicated that these genes are primarily involved in DNA repair, damage checkpoints, response pathways, and p53-mediated signal transduction (Figure 2A).

## 4 Discussion

This study underscores the impact of occupational radon exposure on genomic stability in male iron miners from Shandong, China. Radon, a naturally occurring radionuclide, emits high linear energy transfer (LET) alpha particles during decay (16). In enclosed spaces such as mines and basements, radon accumulates, leading to internal exposure through inhalation (17). It is the second leading cause of lung cancer (18). Despite extensive research on radon’s link to lung cancer, its effects on DNA damage and molecular mechanisms are less explored. Our findings show that radon exposure increases DNA damage in miners’ lymphocytes, as indicated by higher levels of CA and CBMN. These results are consistent with previous studies showing significant DNA damage linked to radon exposure.

DNA damage among underground miners likely results from direct oxidative stress and impaired DNA repair, leading to genomic instability (19, 20). The rise in CA and CBMN levels correlates with longer exposure durations, clearly indicating adverse genetic effects. Miners with over 20 years of exposure showed significantly higher CA and CBMN levels compared to those with 10 years or less, establishing a strong association between exposure duration and cytogenetic damage. Additionally, increased CA and CBMN levels with age suggest that prolonged exposure to high concentrations of radon elevates health risks for miners. In the exposed group, no significant differences in chromosomal aberration or micronucleus rates were found between smokers and non-smokers (Figures 1A,B). Our results indicate that smoking does not interact with radon exposure among underground miners, possibly be related to the small number of statistical samples. Previous studies have also shown the existence of a synergistic effect between radon exposure and tobacco smoke. Both of tobacco smoke and radon generate reactive oxygen species (ROS) that interact with DNA, thought hydroxyl radical attack and radiolysis, leading to saturation of DNA repair pathway and increased apoptosis (21).

Figure 2 illustrates the gene expression changes associated with DNA damage in underground miners exposed to radon, analyzed using the RT^2^ Profiler PCR array. Compared to the reference group, 14 genes were upregulated (*ATM*, *ATR*, *ATPX*, *RAD1*, *RAD18*, *PMS1*, *XRCC2*, *FANCD2*, *MLH3*, *HUS1*, *TP73*, *XPA*, *BARD1*, and *CCNH*), while four genes were downregulated (*BAX*, *DDIT3*, *BBC3*, and *CDC25C*). GO analysis indicated that these genes are primarily linked to DNA repair, DNA damage checkpoints, and DNA damage response pathways. In healthy cells, cell cycle checkpoints maintain a balance between cell proliferation and apoptosis (22). Recent studies have shown that radon exposure and its decay products are directly associated with early lung pathology, immune system suppression, and cellular damage. In our cohort of underground miners exposed to relatively low radon levels and with less occupational co-pollutants, we showed that radon exposure increases genetic instability in underground miners’ lymphocytes, as indicated by higher levels of CA and CBMN. Radon causes DNA damage and high genomic tumor instability, our study supports other findings that low-level, protracted radon exposure causes lung cancer in underground miners. These findings suggest that long-term radon exposure disrupts genomic stability in miners, leading to higher rates of CAs and CBMN, ultimately increasing the risk of lung cancer.

## 5 Conclusion

In conclusion, our study found a strong link between prolonged radon exposure and genomic instability, indicating health risks for underground miners from chronic low-level ionizing radiation. Thus, further measures to reduce radon exposure in mines are critical. Health education for miners and improved management policies should be prioritized to address radon’s health impacts. Additionally, regular monitoring of miners’ workflows and protective measures is recommended to limit radon exposure and support occupational health.

## Data Availability

The raw data supporting the conclusions of this article will be made available by the authors, without undue reservation.

## Footnotes

^1^ www.biocloud.net
